# Supplementary material for: Identification of Important Factors Affecting Use of Digital Individualised Coaching and Treatment of Type 2 Diabetes in General Practice: A Qualitative Feasibility Study
Source: Int J Environ Res Public Health. 2021 Apr 8;18(8):3924. doi: 10.3390/ijerph18083924 (PMC8068375; doi:10.3390/ijerph18083924)
Supplement: Supplementary file 1 [file ijerph-18-03924-s001.pdf]

Supplementary material

Textbox 1:

The digital individualized coaching and lifestyle treatment intervention in DICTA:

1. Individualized digital coaching of T2D patients via an mHealth app.
At inclusion patients install the LIVA app on their smartphone and book a first meeting with their personal health coach (dietician, nurse, occupational therapist, or physiotherapist). The meeting is an online motivational interview, which lasts approximately one hour. Throughout the 12 months intervention period, patients receive personalized relation-driven asynchronous coaching on healthy lifestyle via the Liva app based on individual patient preferences (e.g. increased physical activity, healthier diet, and weight loss) and patient registered outcomes (PRO) data. The preferences are identified in the initial meeting and adjusted throughout the twelve months. The PRO data consists of e.g. steps, diet, and lifestyle plans in relation to the agreed individual goals that the coaches gain access to online. The app also supports the coaches. For example, if a patient's activity level drops, the program recognizes the pattern and advises the coach to trigger actions that will prompt and motivate the patient to achieve the goal. The asynchronous coaching sessions take place once a week in the first 3 months, and every other week for the next 9 months. These are based on dialog by means of text or video. In addition, LIVA is set up to remind patients, e.g. to register lifestyle data. The digital lifestyle intervention is also described in Brandt et al., 2020 [29].

2. Presentation of patient reported outcome (PRO) data to GPs and PNs
PRO data on physical activity, steps, diet, goal fulfilment etc. are provided for the GPs and PNs by linking data registered by the patient or collected by the patient's smartphone through LIVA to the electronic general practice health journal. PRO data will be available for general practice at each patient visit through the electronic health journal. This facilitates doctor-patient discussions on progress in lifestyle changes and impact on T2D treatment based on real time patient data.

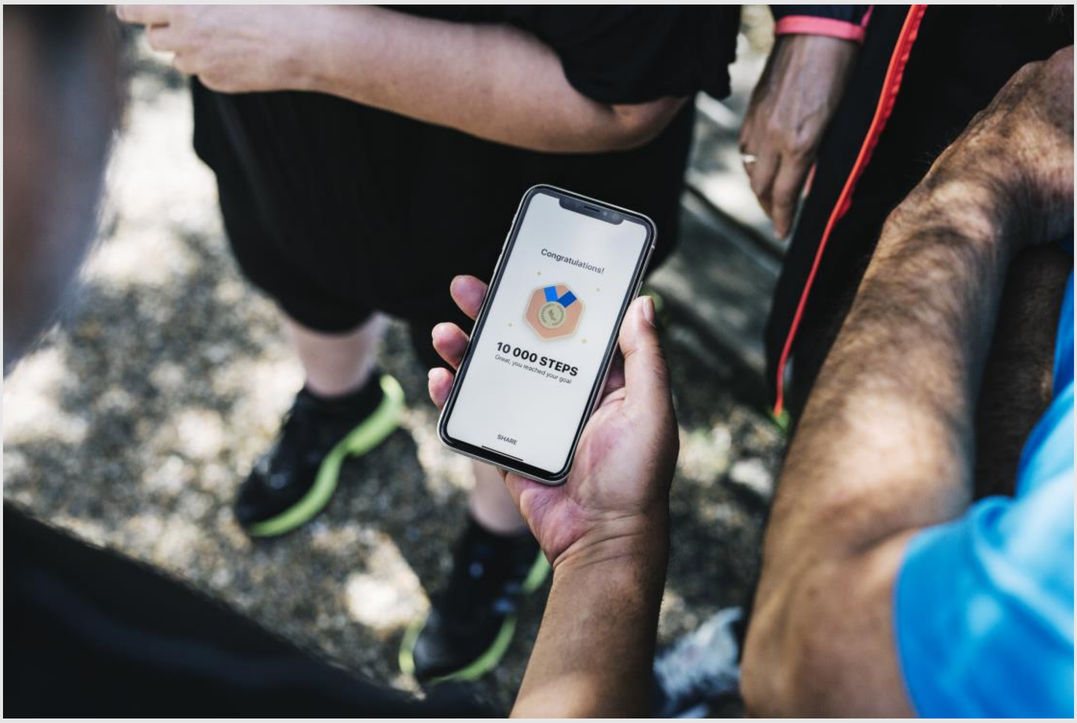

Photo of the Liva app being used to count the daily steps.
